# Effect of modulation factor and low dose threshold level on gamma pass rates of single isocenter multi‐target SRT treatment plans

**DOI:** 10.1002/acm2.14459

**Published:** 2024-07-25

**Authors:** Elena Timakova, Sergei F. Zavgorodni

**Affiliations:** ^1^ University of Victoria Victoria British Columbia Canada; ^2^ BC Cancer Agency Vancouver Island Centre Victoria British Columbia Canada

**Keywords:** AAA, dosimetry, gamma analysis, GPR, modulation factor, Monte Carlo, patient specific QA, plan complexity, SIMT SRT, SRS MapCHECK, verification, VMAT, VMC++

## Abstract

**Purpose:**

SRS MapCHECK (SMC) is a commercially available patient‐specific quality assurance (PSQA) tool for stereotactic radiosurgery (SRS) applications. This study investigates the effects of degree of modulation, location off‐axis, and low dose threshold (LDT) selection on gamma pass rates (GPRs) between SMC and treatment planning system, Analytical Anisotropic Algorithm (AAA), or Vancouver Island Monte Carlo (VMC++ algorithm) system calculated dose distributions.

**Methods:**

Volumetric‐modulated arc therapy (VMAT) plans with modulation factors (MFs) ranging from 2.7 to 10.2 MU/cGy were delivered to SMC at isocenter and 6 cm off‐axis. SMC measured dose distributions were compared against AAA and VMC++ via gamma analysis (3%/1 mm) with LDT of 10% to 80% using SNC Patient software.

**Results:**

Comparing on‐axis SMC dose against AAA and VMC++ with LDT of 10%, all AAA‐calculated plans met the acceptance criteria of GPR ≥ 90%, and only one VMC++ calculated plan was marginally outside the acceptance criteria with pass rate of 89.1%. Using LDT of 80% revealed decreasing GPR with increasing MF. For AAA, GPRs reduced from 100% at MF of 2.7 MU/cGy to 57% at MF of 10.2 MU/cGy, and for VMC++ calculated plans, the GPRs reduced from 89% to 60% in the same MF range. Comparison of SMC dose off‐axis against AAA and VMC++ showed more pronounced reduction of GPR with increasing MF. For LDT of 10%, AAA GPRs reduced from 100% to 83% in the MF range of 2.7 to 9.8 MU/cGy, and VMC++ GPR reduced from 100% to 91% in the same range. With 80% LDT, GPRs dropped from 100% to 42% for both algorithms.

**Conclusions:**

MF, dose calculation algorithm, and LDT selections are vital in VMAT‐based SRT PSQA. LDT of 80% enhances sensitivity of gamma analysis for detecting dose differences compared to 10% LDT. To achieve better agreement between calculated and SMC dose, it is recommended to limit the MF to 4.6 MU/cGy on‐axis and 3.6 MU/cGy off‐axis.

## 1 INTRODUCTION

Radiotherapy is a longstanding and effective method for treatment of cancers, with the aim of maximizing dose delivered to target volume and minimizing dose delivered to normal tissues. Stereotactic radiosurgery (SRS) takes this approach further by utilizing highly conformal dose distributions with steep dose gradients effectively delivering high therapeutic prescription doses to tumor tissues and low doses to surrounding tissues.^1^ Brain metastases are the most common intracranial tumor occurring in 30% of cancer patients.^2^ SRS and SRT can be used to treat single or multiple lesions, including those surgically inaccessible.^3^ SRS is an accepted form of treatment for patients with up to three metastasis,^4^, ^5^ although some studies demonstrated its feasibility for five or more metastasis.^6^, ^7^ Multi‐met cases are frequently planned using multitarget single isocenter (MTSI) technique, where the isocenter is placed between targets, to reduce treatment time and costs.^8^, ^9^ Targets can range anywhere from 0.7 to 30 cc^10^ with distances up to 10 cm from isocenter.^11^

In SRS and SRT one of the most commonly used radiation delivery techniques is volumetric‐modulated arc therapy (VMAT). VMAT is able to rapidly deliver highly conformal doses by simultaneous modulation of gantry rotation speed, multi‐leaf collimator (MLC) position, and dose rates.^12^, ^13^ Although, beam intensity modulation allows optimized dose delivery, high degrees of complexity result in larger dosimetric uncertainty, leading to disagreement between planned dose and delivered dose. Highly modulated plans increasingly use small and irregular beams, where calculation accuracy of current algorithms is limited,^14^, ^15^ while more complex mechanical movements of gantry and MLC increase mechanical uncertainty.^16^, ^17^, ^18^ Increased complexity of VMAT delivery necessitates pre‐treatment dose verification, commonly performed via pre‐treatment patient‐specific quality assurance (PSQA).

The most popular analysis metric for PSQA is the gamma index as proposed by Low et al. (1998). Gamma index evaluates the agreement of measured dose to calculated dose by using percent point dose difference (%DD) and distance to agreement (DTA) criteria.^19^, ^20^ American Association of Physicists in Medicine (AAPM) Task Group 218 (TG‐218),^21^ recommends using global gamma index analysis to calculate %DD, where %DD is calculated relative to the maximum (prescription) dose. AAPM TG‐218 also recommends using 2 mm/3% (DTA/%DD) criteria with gamma passing rates (GPRs) over 95% calculated in absolute dose mode with low dose threshold (LDT) of 10%. TG‐218 indicates that tighter tolerances should be considered for SBRT and SRT cases, although does not allude to specific values. Several studies have found that 2 mm/3% is insufficient to detect errors in VMAT and tighter tolerances like 1 mm/3% with GPRs ≥ 90% should be used, especially for SRT.^22^, ^23^, ^24^, ^25^ Several studies report LDT of 10% is not sensitive enough in selecting the region of evaluation.^26^, ^27^ Few studies evaluate GPRs with LDT above 10%, but Lu et al. showed higher accuracy of detecting dose errors when using LDT encompassing the planning target volume (PTV) and organs at risk (OAR) than the conventional 10%.^28^ A significant factor affecting GPR is plan complexity, where higher complexity plans have higher beam delivery uncertainty resulting in lower GPRs.

SRS MapCHECK (SMC) is a diode array designed to perform PSQA particularly for SRS/SRT cases. This device has previously been validated for general dosimetric characteristics such as dose‐response linearity, dose rate, field size, and angular dependence.^29^, ^30^, ^31^ Rose et al. evaluated the gamma pass rates (GPRs) (3%/1 mm) between SMC and film or treatment planning system (TPS) for 84 SRT or SBRT plans with varying modulation factors (MFs) and reported failure at the highest MFs of 4.9 and 5.5 MU/cGy. Rose et al. recommended limiting MFs to 3 MU/cGy for good agreement between methods.^32^ These results were echoed by Lee and Kim who reported lower GPRs (2%/1 mm criteria) of volumetric modulated arc therapy (93.71%) plans compared to dynamic conformal arc therapy (99.54%) plans as a result of higher modulation in VMAT plans due to the nature of the techniques.^33^

Previous research with SMC has explored basic dosimetric characteristics, but limited data is available for off‐axis positions and high degrees of modulation, and the selection of gamma criteria has raised concerns that require careful consideration. Consequently, the authors conducted further investigation into the effects of degree of modulation on GPRs between SMC and treatment planning system TPS or Monte Carlo (MC) dose calculations. By utilizing two types of calculation algorithms, they aimed to explore a more comprehensive correlation between GPRs of SMC and calculated dose via algorithm. Additionally, the relevance of LDT selection for gamma index analysis was investigated. Finally, the authors sought to examine the relationship between GPRs and location of PTV at the isocenter compared to off‐axis position to mimic SIMT SRT.

## 2 METHODS

### 2.1 Treatment plans

Single‐isocenter‐multi‐target (SIMT) SRT is commonly employed for treating multiple brain metastases, literature suggests that majority of these metastases exhibit a roughly spherical shape.^34^ Consequently, a near‐spherical PTV serves as a suitable representation for the majority of cases. The PTV dimensions were chosen to be around 2 cm in diameter to be representative of a potential SIMT SRT treatment and to ensure that the field size was not inherently too small in order to reduce effects of small field conditions. The study comprises of two sets of plans: the first set features PTV located at the isocenter, while the second set features PTV situated off‐axis. This simplified model is designed to encompass a range of scenarios encountered in SIMT SRT cases, while minimizing confounding variables such a variability in tumor size, inhomogeneity considerations, small field effects, and similar.

#### 2.1.1 Modulation factors

There are various definitions of MF in the literature. In this paper MF was defined in its simplest form as the number of monitor units (MUs) divided by the central PTV dose as calculated by the TPS (Eclipse AAA Version 13.6.23).

#### 2.1.2 On‐axis treatment plans

A set of 16 co‐planar on‐axis plans was created in a phantom with MFs ranging from 2.66 to 10.21 MU/cGy. This study was intentionally conducted as a phantom study to minimize confounding variables by maintaining consistent PTV and “patient” geometry. To force optimization algorithms to produce plans with different degree of modulation, PTV coverage constraints and artificial dose constraint structures were employed. Requirement for the algorithm to use a set number of MUs was also used to achieve required modulation level. The jaws were set to 4 × 4 cm^2^. By keeping the field at this larger size, any effects of the jaw scatter will be reduced and observed trends should be due to modulation alone. The on‐axis scenario was representative of one of the targets located on‐axis in a SIMT SRT plan, or a single target SRT plan. Some of the plans were created with the use of Progressive Resolution Optimizer (Version 13.6.23) and some were created with the use of Photon Optimizer (Version 15.6.06).

#### 2.1.3 Off‐axis treatment plans

A set of 11 co‐planar plans was produced off‐axis. In this case, PTV was shifted +6 cm in *Z*‐direction (DICOM patient coordinate system) relative to isocenter, and plans were re‐optimized using the same optimization parameters as for isocentric plans. The off‐axis scenario was representative of one of the targets located off‐axis in a SIMT SRT plan. This scenario was designed to evaluate the performance of SMC dosimetry in off‐axis situations. The MF ranged from 2.70 to 9.76 MU/cGy.

All plans were created in Eclipse system by Varian Medical Systems Inc (Palo Alto, CA, USA). The doses were calculated with AAA (1 mm resolution) and Monte Carlo VMC++ (1 mm resolution, 1% uncertainty) algorithms.

### 2.2 SRS MapCHECK

This dosimetry system consists of three components including the SMC diode detector array, the StereoPHAN phantom, and the SNC Patient Software, all provided by Sun Nuclear Corporation, Melbourne, FL, USA.

SMC is a diode array with 1013 SunPoint 2 diode (active area of 0.48 × 0.48 mm^2^ and an active volume of 0.007 mm^3^) detectors. The diodes are arranged on two printed circuit boards, shifted relative to each other by 1.75 mm in *X* and *Y* directions, covering a total area of 77 × 77 mm^2^. The spacing between diodes is 2.47 mm at 45° and in‐line spacing between diodes is 3.50 mm, resolution of array is 2.47 mm. Both, the build‐up and the backscatter plates consist of 22 mm thick polymethyl methacrylate (PMMA) plates.

StereoPHAN is designed to house the SMC for end‐to‐end testing of SRS plans. The StereoPHAN is a cylindrical phantom with a hemispherical end to mimic the head. Its diameter is 152 mm, its length is 208 mm, and it is composed of PMMA. The phantom offers five fiducial markers to help with alignment. The cylinder can rotate a full 360°.

SNC Patient Software is a quality assurance software that works in conjunction with SMC to calibrate the array, to measure dose, and to analyze dose distributions. This software calculates and applies all relevant correction factors to the diodes to convert raw readings to a dose measurement. The software provides comparisons between measured and planned dose distributions or absolute to relative doses via gamma analysis, based on user selected values.

The raw diode response is converted to absolute dose with the use of calibration and correction factors obtained during the calibration procedure and applied via the SNC Patient Software (version 8.4). SNC Patient Software allows implementation of corrections for angle of beam incidence, couch angle, temperature, small fields, and dose rate. The absolute dose for a given diode takes into account the background counts, the calibration dose, and correction factors.

### 2.3 Dose calculation algorithms

#### 2.3.1 TPS algorithm—Analytical Anisotropic Algorithm (AAA)

Analytical anisotropic algorithm (AAA) for photon dose calculations was developed by Varian Medical Systems, Palo Alto, CA, USA, and works of Ulmer et al., and has been described extensively in literature,^35^, ^36^, ^37^, ^38^, ^39^, ^40^, ^41^, ^42^ thus only a brief description is presented here. AAA consists of a configuration module, which characterizes the phase space for a given linear accelerator beam, and a dose calculation module, which calculates the total dose through superposition of dose deposited by primary photons, extrafocal photons, and contamination electrons. To calculate dose to the patient, the beam is subdivided into beamlets. Dose contributions from a beamlet are defined by separate convolution functions for each component, attenuation is defined by energy deposition density functions, while lateral scatter is defined by scatter kernels. The total dose for each voxel is defined by the superposition of dose contributions from each beamlet.

All TPS calculations were performed on the phantom CT image dataset of SMC provided directly by SunNuclear Corporation with a grid size of 1 mm.

#### 2.3.2 Vancouver Island Monte Carlo

Vancouver Island Monte Carlo (VIMC) is a system developed by a team at Vancouver Island Cancer Center, Victoria, BC, Canada, for verification of existing treatment plans.^43^, ^44^ A set of files including CT, plan, and dose files are exported from a TPS and imported into VIMC to perform fully automated patient dose calculations.^45^ Currently, Varian Clinac 21EX or Varian TrueBeam can be modelled with BEAMnrc and phase space files provided by Varian Medical Systems,^45^ with latent variance reduction techniques such as azimuthal particle redistribution.^46^ VIMC allows selection of jaw and MLC models (rigorous or fast models) and dose calculation codes (DOSXYZnrc or VMC++). This work used a combination of fast dose calculation code (VMC++), as well as, fast jaw tracking and MLC models. MLC model was developed by Keall et al.^47^ and Siebers et al.,^48^ and jaw model was developed and reported by Townson et al.^49^ Once all files are imported, a patient volume is created via DOSXYZnrc CTcreate code, and converted to binary for use with VMC++. The user sets an uncertainty and the number of required histories is calculated. For plans with multiple beams, uncertainty for each beam is added in quadrature.^45^ Then patient dose is calculated using VMC++, which is well described in literature.^50^, ^51^, ^52^, ^53^, ^54^ In summary, VMC++ is a MC simulation of coupled photon‐electron transport optimized for treatment planning dose calculation for radiotherapy applications. Due to all of the variance reduction techniques employed, VMC++ is one of the fastest and most accurate MC codes for particle transport and dose calculation currently available. Dose output by VMC++ is given in normalized units of Gy/electron, thus conversion to absorbed dose units of Gy is performed via established relationship between dose in the monitor ionization chamber of the linac, the number of particles incident on the target, and the field size.^55^ Finally, the dose distributions are converted to DICOM to be imported into the TPS.

All VMC++ calculations were performed with voxel size of 1 mm and uncertainty of 1%.

### 2.4 Measurements

All measurements were performed with Varian TrueBeam STx linear accelerator (Varian Medical Systems, Palo Alto, CA, USA) equipped with a high definition MLC—leaves are 2.5 mm wide at isocenter. All plans were delivered to SMC with 6 MV with flattening filter (6 MV‐WFF) energy beam. SMC phantom was positioned such that central diode of the SMC was at the center of the PTV.

Part of calibration procedure for SMC was taking a known dose measurement. This accounted for daily machine output variations. All off‐axis plans were delivered on the same day, and this same day two thirds of isocentric plans were delivered. One third of isocentric plans was delivered on a day prior.

The measurements were compared against doses calculated with AAA (1 mm voxels) and VMC++ (1 mm voxels, 1% uncertainty). SNC Patient software was used to evaluate GPRs.

### 2.5 Gamma analysis metrics

The gamma index measures coincidence between measured and calculated dose distributions using %DD and distance‐to‐agreement (DTA) criteria. Global gamma, relative to maximum dose, with LDTs of 10% to 80% were evaluated. The threshold of 10% was chosen because it is recommended in AAPM TG 119 report for IMRT gamma analysis,^56^ and is used in many publications. The upper value of 80% was chosen because this is a “classical” SRT dose prescription value and it is also close to the volume of “100% isodose covering 95% of PTV” as prescribed in many modern VMAT based SRT protocols. With a focus on SRT applications, DTA tolerance was chosen at 1 mm following clinical protocol, and %DD was chosen at 3%. GPR calculated via SNC Patient software was reported. GPRs below 90% were considered as fail, thus acceptance criteria was GPR of 90%. These gamma analysis criteria are consistent with recent publications on SRS/SRT QA.^22^, ^24^, ^25^, ^57^

## 3 RESULTS

### 3.1 On‐axis VMAT dose distributions

**FIGURE 1:**
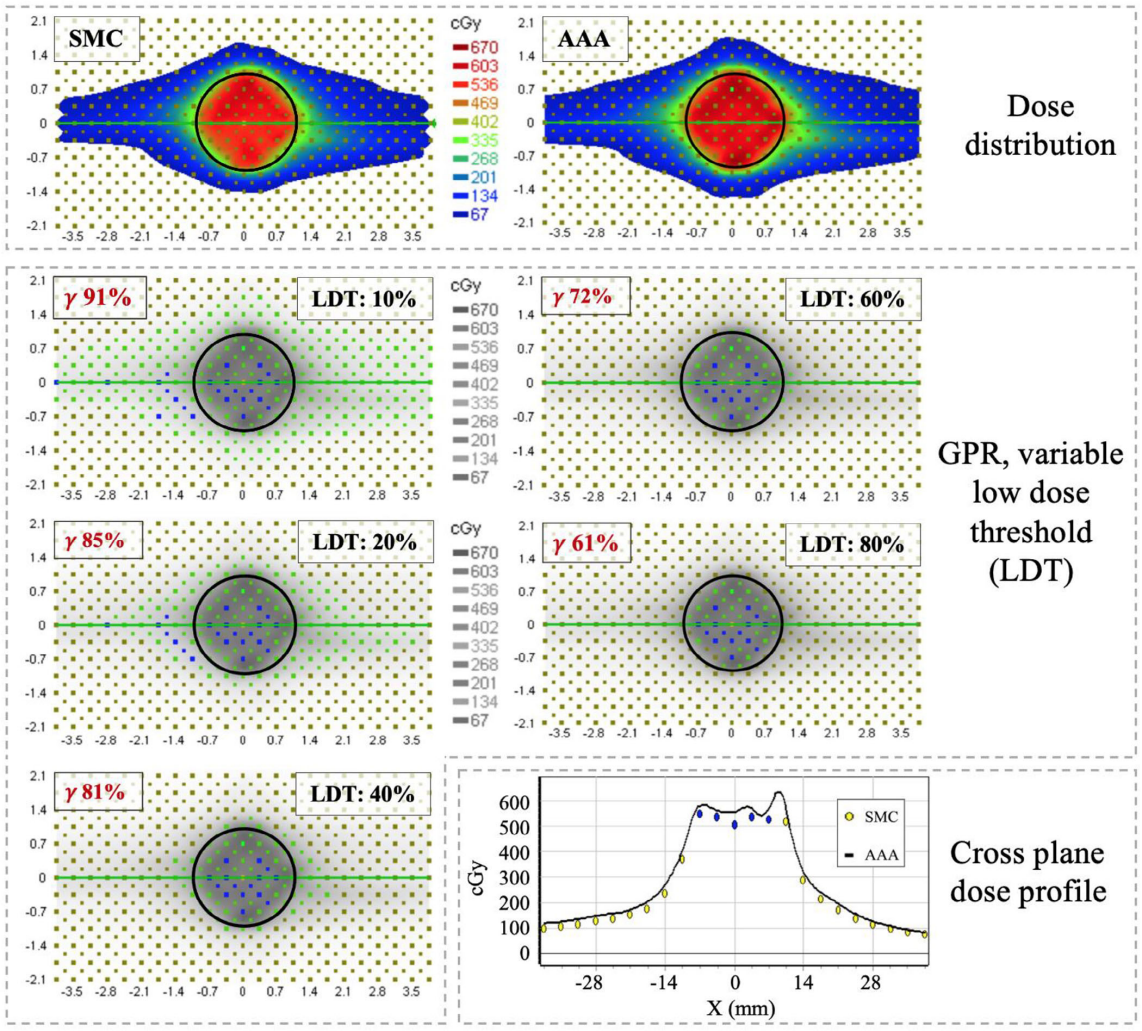
Analysis, with SNC patient software, of the dose distributions produced by SMC and AAA for an isocentric VMAT plan with MF = 8.5 MU/cGy. Top panel: Dose distributions of SMC (left) and AAA (right). Middle panel: Diodes, displaying pass/fail as green/blue, respectively, as well as, GPR with LDT of 10%, 20%, 40% (left) and 60%, 80% (right). Black circles are approximate PTV (d = 2 cm). Bottom panel: Cross‐plane dose profile through central diode. AAA—solid line, SMC—circles, yellow circles indicate pass, blue circles indicate fail.

Figure 1 displays gamma analysis comparison of a plan with MF of 8.5 MU/cGy, demonstrating the differences in GPR between an LDT of 10%, 20%, 40%, 60%, and 80%. Top panel displays the dose distributions reconstructed by SMC (left) and calculated by AAA (right) in color gradient. Middle panel displays the gamma analysis with grey‐scale dose color‐wash. The grey dots indicate diode positions. Colored dots indicate diodes used for gamma analysis, with green meaning pass, blue meaning fail with SMC dose below %DD threshold. The black circle outline roughly shows a 2 cm PTV boundary. GPR for 10% LDT was 91%, comfortably above the GPR ≥ 90% acceptance criteria, while GPR for lower LDT values steadily decreased with increasing LDT. GPR for LDT of 80% was below acceptance criteria being only 61%. Note many passing diodes were located well outside of 2 cm PTV boundary when LDT of 10% was used, boosting GPR. In contrast, with 80% LDT, just the diodes within the PTV region were evaluated, effectively providing a GPR for the region of interest, around prescription isodose level. Similar results were seen for other values of MF. Bottom panel shows cross‐plane dose profile reconstructed by SMC against that calculated by AAA.

**FIGURE A1:**
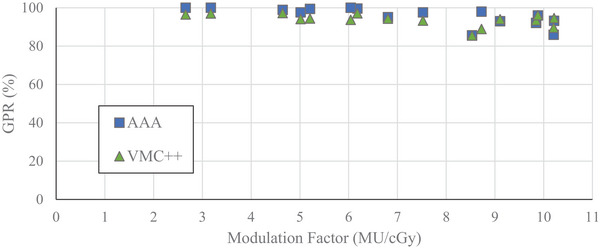
Gamma pass rates as a function of modulation factor for 6 MV‐WFF energy beam, calculated with 20% low dose threshold for VMAT plans with on‐axis PTV. SMC‐AAA—blue square. SMC‐VMC++—green triangle.

**FIGURE A2:**
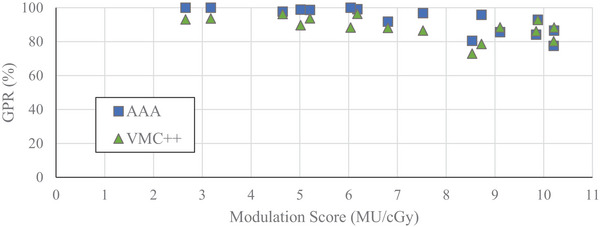
Gamma pass rates as a function of modulation factor for 6 MV‐WFF energy beam, calculated with 40% low dose threshold for VMAT plans with on‐axis PTV. SMC‐AAA—blue square. SMC‐VMC++—green triangle.

**FIGURE A3:**
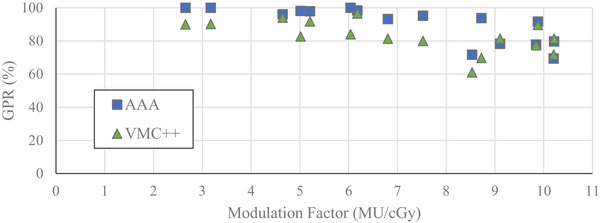
Gamma pass rates as a function of modulation factor for 6 MV‐WFF energy beam, calculated with 60% low dose threshold for VMAT plans with on‐axis PTV. SMC‐AAA—blue square. SMC‐VMC++—green triangle.

**FIGURE A4:**
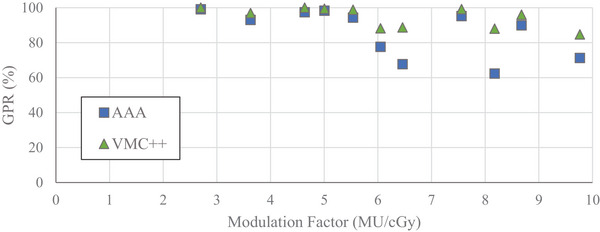
Gamma pass rates as a function of modulation factor for 6 MV‐WFF energy beam, calculated with 20% low dose threshold for VMAT plans with off‐axis PTV. SMC‐AAA—blue square. SMC‐VMC++ —green triangle.

**FIGURE A5:**
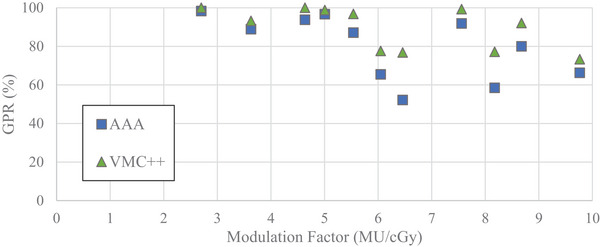
Gamma pass rates as a function of modulation factor for 6 MV‐WFF energy beam, calculated with 40% low dose threshold for VMAT plans with off‐axis PTV. SMC‐AAA—blue square. SMC‐VMC++ —green triangle.

**FIGURE A6:**
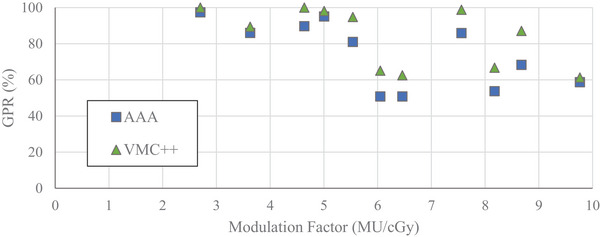
Gamma pass rates as a function of modulation factor for 6 MV‐WFF energy beam, calculated with 60% low dose threshold for VMAT plans with off‐axis PTV. SMC‐AAA—blue square. SMC‐VMC++ —green triangle.

Due to the considerations concerning the distribution of passing diodes with respect to the 2 cm PTV region and their consequential influence on GPR, the main text of this paper presents results for LDT values of 10% and 80%. Results for other LDT values are provided in the Appendix for completeness, see Figures A1, A2, A3, A4, A5, A6.

**FIGURE 2:**
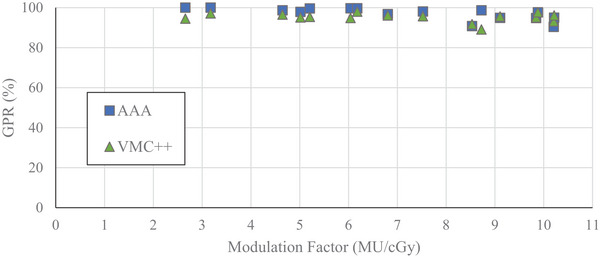
Gamma pass rates as a function of modulation factor for 6 MV‐WFF energy beam, calculated with 10% LDT for VMAT plans with on‐axis PTV. SMC‐AAA—blue square. SMC‐VMC++ —green triangle.

**FIGURE 3:**
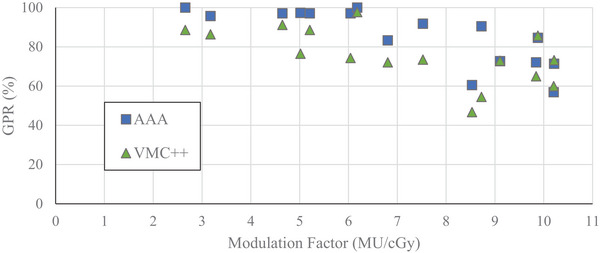
Gamma pass rates as a function of modulation factor for 6 MV‐WFF energy beam, calculated with 80% LDT for VMAT plans with on‐axis PTV. SMC‐AAA—blue square. SMC‐VMC++ —green triangle. AAA, Analytical Anisotropic Algorithm; PTV, planning target volume; SMC, SRS MapCHECK; VMAT, volumetric‐modulated arc therapy; VMC, Vancouver Island Monte Carlo.

The GPRs (with 3%/1 mm criteria) between SMC and computationally derived dose distributions for VMAT plans delivered on‐axis as a function of MF are found in Figures 2 and 3. MFs ranged from 2.66 to 10.21 MU/cGy. Generally, a negative correlation between MF and GPR was observed.

With a low dose threshold of 10%, a plateau region with consistent GPR was evident up to MF of 6.2 MU/cGy (Figure 2). The average GPRs in this region were 99% and 96% for SMC comparison to AAA and VMC++, respectively. For MFs higher than 6.2 MU/cGy, a small reduction in GPR was observed with an average of 95% and 94% for AAA and VMC++, respectively. With LDT of 10%, 100% of AAA calculated plans and 99% of VMC++ calculated plans were within acceptance criteria of GPR ≥ 90% when compared to SMC.

Similar, but more pronounced trends were observed when using a low dose threshold of 80% (Figure 3). In this case, the plateau region was evident up to 6.2 and 4.6 MU/cGy when comparing SMC to AAA and VMC++, respectively, and GPRs reduced with higher MFs. The average GPRs in this region were 98% and 89% for SMC comparison to AAA and VMC++, respectively. For higher MFs, the GPRs dropped off significantly to an average of 76% and 72% for SMC comparison to AAA and VMC++, respectively. Considering GPRs with 80% LDT over the entire range of MFs, SMC agreed with AAA or VMC++ within acceptance criteria of GPR ≥ 90% in 56% and 13% of these on‐axis VMAT cases, respectively.

**FIGURE 4:**
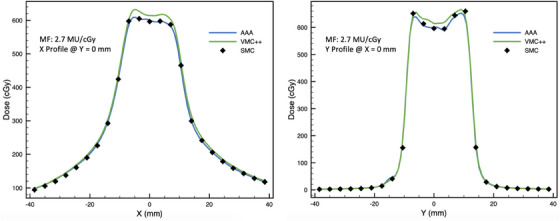
Cross‐plane (left panel) and in‐plane (right panel) profiles through the central diode for on‐axis VMAT plan with modulation factor of 2.7 MU/cGy (with 10% LDT, GPRs for AAA and VMC++ are 100% and 95%, respectively; with 80% LDT GPRs for AAA and VMC++ are 100% and 87%, respectively).

**FIGURE 5:**
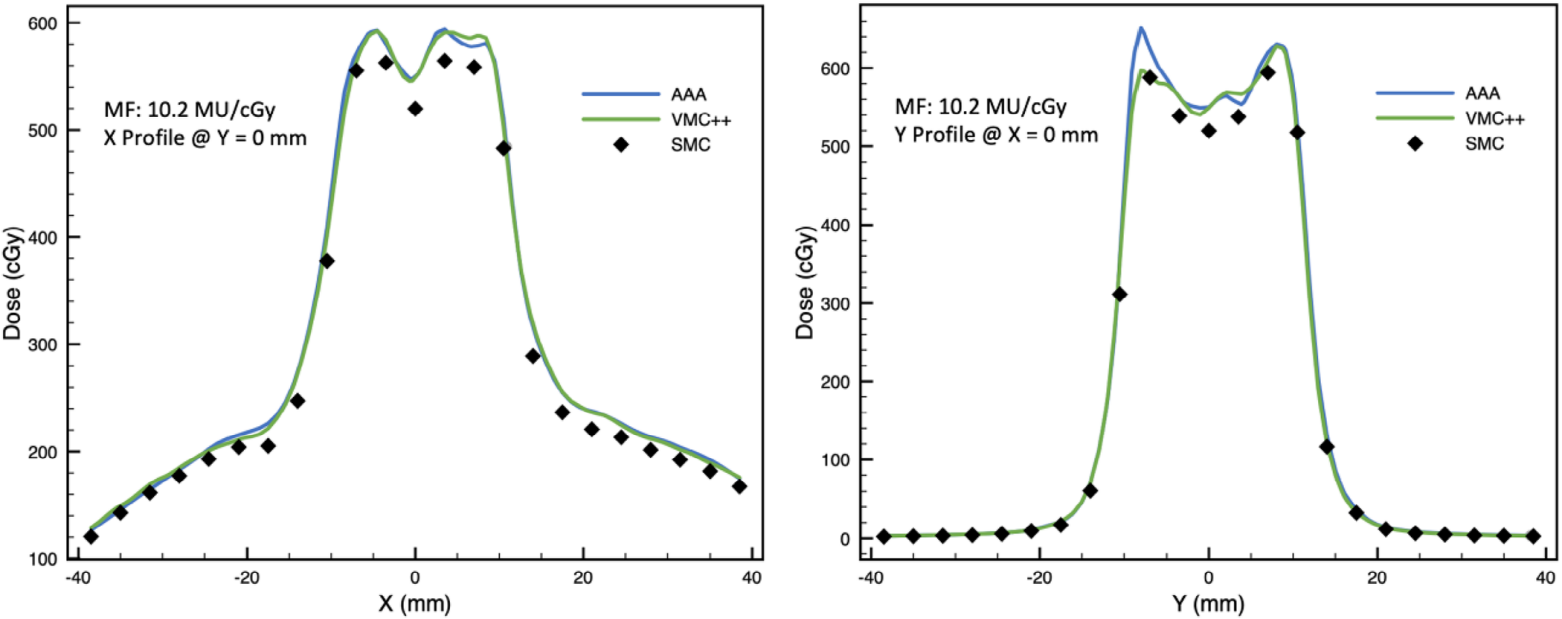
Cross‐plane (left panel) and in‐plane (right panel) profiles through the central diode for on‐axis VMAT plan with modulation factor of 10.2 MU/cGy (with 10% LDT GPRs for AAA and VMC++ are 90% and 93%, respectively; with 80% LDT GPRs for AAA and VMC++ are 57% and 60%, respectively).

Cross‐plane and in‐plane profiles for the highest and lowest GPRs for on‐axis VMAT plans are found in Figures 4 and 5.

### 3.2 Off‐axis VMAT dose distributions

**FIGURE 6:**
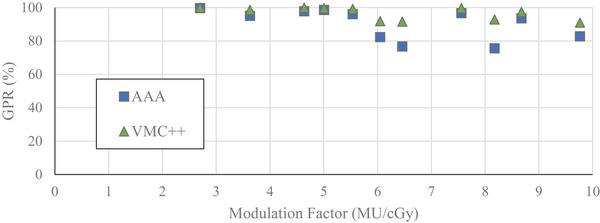
Gamma pass rates as a function of modulation factor for 6 MV‐WFF energy beam, calculated with 10% LDT for VMAT plans with off‐axis PTV. SMC‐AAA—blue square. SMC‐VMC++ —green triangle.

**FIGURE 7:**
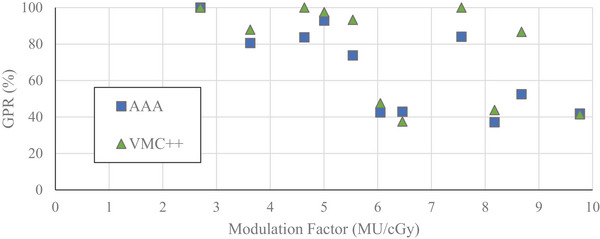
Gamma pass rates as a function of modulation factor for 6 MV‐WFF energy beam, calculated with 80% LDT for VMAT plans with off‐axis PTV. SMC‐AAA—blue square. SMC‐VMC++ —green triangle.

For off‐axis VMAT delivery, GPRs (with 3%/1 mm criteria) as a function of MF are plotted in Figures 6 and 7. MF ranged from 2.70 to 9.76 MU/cGy. Overall, a negative correlation between MF and GPR was seen. As seen in Figure 6, for LDT of 10%, a plateau region was evident up to 5.5 MU/cGy with average GPRs of 98% and 100% when SMC was compared to AAA and VMC++, respectively. A reduction in GPR was noted for MFs above 5.5 MU/cGy with average values of 85% and 94% comparing SMC to AAA and VMC++, respectively. With 10% LDT, 100% of off‐axis VMAT plans were within acceptance criteria when comparing SMC to VMC++, while 64% of plans were within acceptance criteria when comparing SMC to AAA.

As seen in Figure 7, for LDT of 80%, when comparing SMC to VMC++, a plateau region was evident up to MF of 5.5 MU/cGy with an average GPR of 96%. Comparing SMC to AAA the plateau region was not as evident with an average GPR of 86%. For higher MF region, the average GPR fell to 50% and 60% when SMC was compared to AAA and VMC++, respectively. With 80% LDT, 45% of plans were within acceptance criteria when comparing SMC to VMC++ and 18% of plans were within acceptance criteria when comparing SMC to AAA.

**FIGURE 8:**
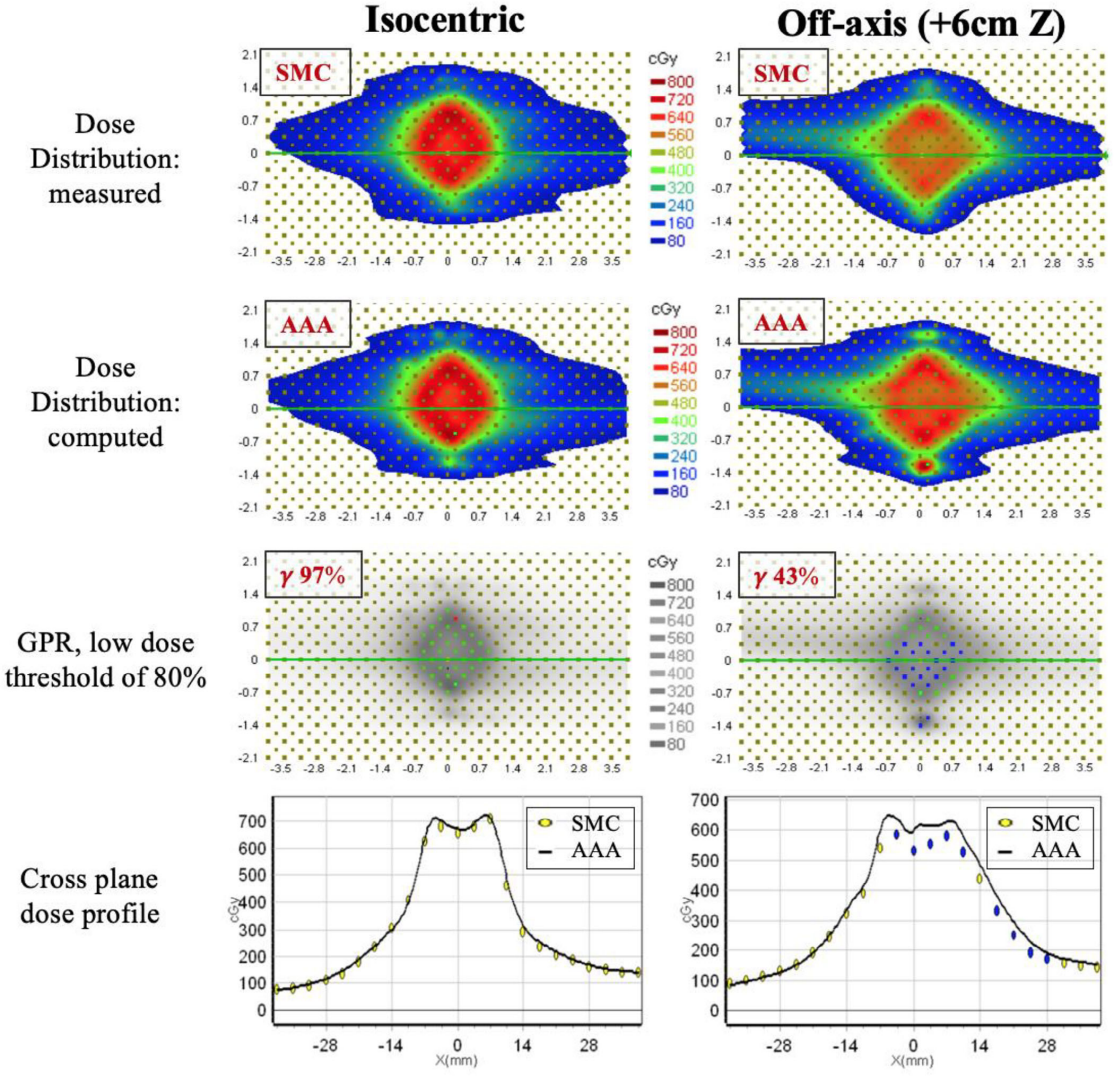
Analysis, utilizing the SNC Patient software, of the dose distributions produced by SMC and AAA for isocentric and off‐axis VMAT plans. Top panel: Dose distributions reconstructed by SMC for isocentric (left) and off‐axis (right) plans. Second panel: Dose distributions calculated by AAA for isocentric (left) and off‐axis (right) plans. Third panel: Diodes, displaying pass/fail as green/blue, respectively, as well as, GPR calculated for isocentric (left) and off‐axis (right) plans. Bottom panel: Cross‐plane dose profile through central diode. (AAA—solid line, SMC—circles. Yellow circles indicates pass, blue circles indicates fail).

Figure 8 displays gamma analysis comparison of isocentric and off‐axis VMAT plans with the same MF of 6.0 MU/cGy. With LDT of 80% the GPR of AAA calculated isocentric plan was 97%, well within acceptance criteria of GPR ≥ 90%, while GPR for the off‐axis plan was only 43%.

**FIGURE 9:**
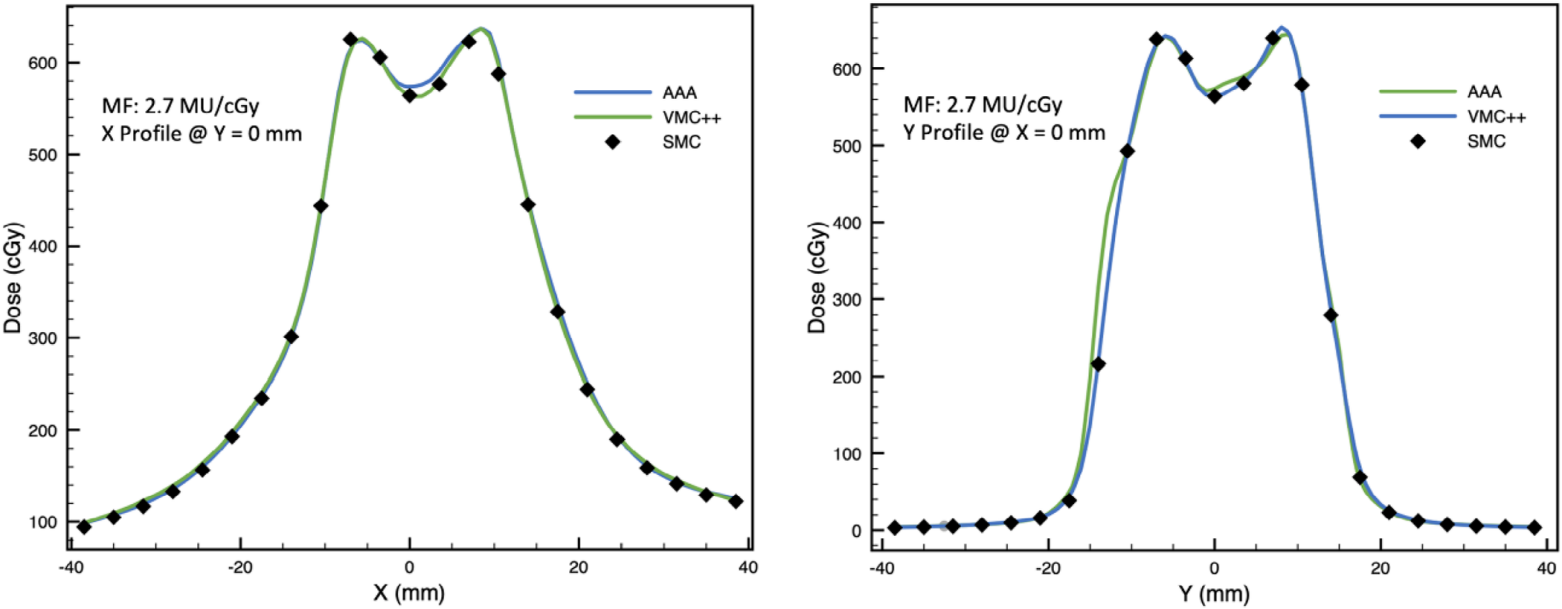
Cross‐plane (left panel) and in‐plane (right panel) profiles through the central diode for off‐axis VMAT plan with modulation factor of 2.7 MU/cGy (with 10% LDT, GPRs for AAA and VMC++ are 100% for both; with 80% LDT, GPRs for AAA and VMC++ are 100% for both).

**FIGURE 10:**
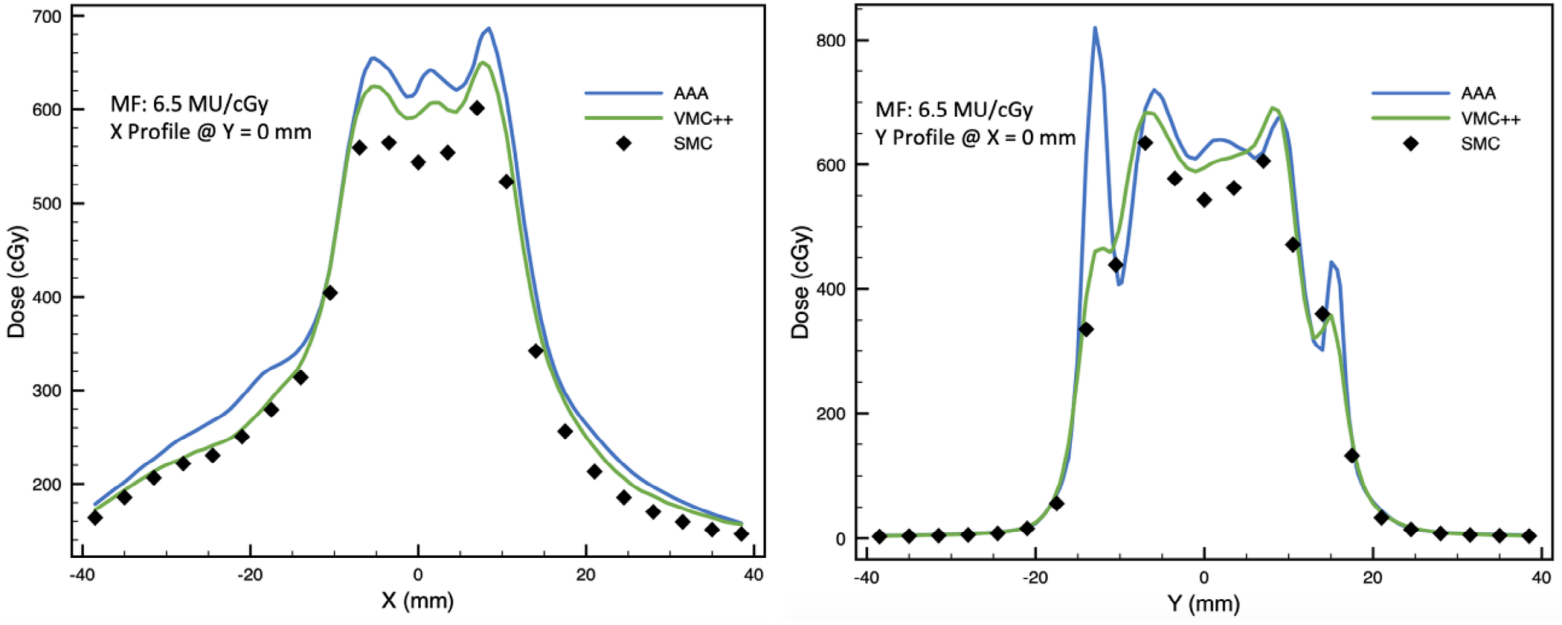
Cross‐plane (left panel) and in‐plane (right panel) profiles through the central diode for off‐axis VMAT plan with modulation factor of 6.5 MU/cGy (with 10% LDT, GPRs for AAA and VMC++ were 77% and 92%, respectively; with 80% LDT, GPRs for AAA and VMC++ were 43% and 38%, respectively).

Cross‐plane and in‐plane profiles for the lowest and highest GPRs associated with off‐axis VMAT plans can be found in Figures 9 and 10. Figure 10 shows considerable disagreement in profiles measured by SMC and calculated with AAA and VMC++. The dose difference is the greatest for AAA at in‐plane profile at around –15 mm and +15 mm away from central diode. In the plan, these regions contain very small MLC openings contributing uncertainty associated with small field dose calculations.

## 4 DISCUSSION

Clinical motivation for using highly modulated VMAT fields is that such fields can considerably improve conformity of the dose and dose fall‐off outside of the PTV. This applies to cases with single PTV, as well as to treatments of multiple metastasis using single isocenter VMAT SRT, where all PTVs are located off‐axis. However, potential plan quality improvement due to high modulation has to be balanced against uncertainties in the dosimetry of highly modulated fields. Such fields typically consist of larger numbers of small and irregular MLC sub‐fields irradiating the target volumes and presenting challenges for accurate dose modeling and measurements. Gamma analysis^19^ is commonly used to quantify differences between calculated and measured dose distributions.

There are many studies on the appropriate action limits, tolerances, and criteria for gamma analysis to be used in IMRT and VMAT treatments. The main recommendation for IMRT QA is given by AAPM TG 218,^21^ which proposes using %DD/DTA of 3%/2 mm with LDT of 10% and GPR of 90%. Tighter tolerances are suggested for SRS/SRT treatments where PTV margins in many clinics are accepted to be as small as 1 mm or less. Although no specific recommendations for intracranial SRS/SRT VMAT are available, many studies recommend tighter tolerances, such as 3%/1 mm with GPRs ≥ 90%.^22^, ^24^, ^25^, ^57^ Additionally, due to high‐dose gradients and small margins prevalent in SRS/SRT VMAT, changes in LDT may be required to adjust the evaluation region.

The correlation between GPR (3%/1 mm) and MF was examined in this study for dose measured with SMC and dose calculated either with Eclipse AAA or VIMC VMC++. The gamma analysis was performed for five LDT settings (10%, 20%, 40%, 60%, and 80%) for a set of SRT VMAT plans with the dose located at isocenter or off‐axis at +6 cm in *Z*‐direction following DICOM coordinate system. With each increase in LDT from 10% to 80%, a decrease in GPR value was observed. This highlights decreasing levels of sensitivity to dose differences via gamma analysis performed with lower LDT values.

For the plans with dose distribution produced near isocenter, GPRs with 10% LDT all passed acceptance criteria of GPR ≥ 90% when comparing SMC to AAA for MFs of 2.7 to 10.2 MU/cGy (Figure 2). Only a single marginal failure was seen between SMC and VMC++ at 8.7 MU/cGy. For GPRs with 80% LDT (Figure 3), GPRs consistently failed above 6.2 MU/cGy comparing SMC to AAA and 4.6 MU/cGy when comparing SMC to VMC++.

As seen in the results of this study, the value of 10% for LDT masks differences in high dose regions as it averages pass rates over high and low dose regions. Figure 1 demonstrates that 10% LDT uses almost 7× more diodes than 80% LDT, with majority of passing diodes located outside the PTV region, resulting in an artificially high GPRs. In the context of multi‐met SRT treatments, dose regions encompassing the target are critical clinically and should be emphasized in gamma analysis metrics, while low dose regions are usually less important.

In our study, GPRs with 10% LDT were consistently high across a wide range of MFs (Figure 2), potentially allowing delivery errors to remain unnoticed. With selection of 10% LDT, plans may well pass the action limit of GPR ≥ 90%, however, with 80% LDT, plans may fail, prompting further analysis and investigation. Examples of these cases are seen when comparing plans with MF exceeding 6.2 MU/cGy in Figures 2 and 3. Therefore, an LDT of 80%, which encompasses the PTV, provides a more selective indication of potential dosimetry failure in the PTV region.

Similar findings were shown by Lu et al, who demonstrated that γ_whole_ (LDT selected such that only PTV and OARs were included) was a superior metric than conventional γ_10_ (LDT of 10%) for detection of small systematic MLC errors and clinical effect of radiotherapy.^28^ Other studies such as Cozzolino et al.^58^ and Strauss and Shaw^59^ suggest using GPR per structure rather than a single GPR for the entire patient geometry, to increase sensitivity of finding potential errors.

Given higher sensitivity of 80% LDT to potential errors, especially in the critical PTV volume, we suggest that an LDT value of 80% is more appropriate for GPR analysis than the commonly reported value of 10%. This LDT value best reflects PTV coverage, which is crucial in most SRT treatments, particularly for metastases. However, it may not include dose areas around OAR. Therefore, in our opinion, it would be beneficial if manufacturers included options for more than one dose evaluation region into the analysis software.

Overall, across all conditions examined in this study, GPR showed a negative correlation with MF. Typically, a plateau region with high GPR was observed for MFs up to about 4.6 to 6.2 MU/cGy. Beyond this region a significant reduction in GPR was observed. Therefore, based on our results, we recommend an upper limit of 4.6 MU/cGy for MFs, as different dosimetry methods are likely to considerably disagree for the plans with higher modulation. This recommendation is further supported by profiles seen in Figures 4 and 5, which demonstrate a good agreement between SMC, AAA, and VMC++ for VMAT plan with low MF (2.7 MU/cGy) and poor agreement for high MF (10.2 MU/cGy). This is in line with findings by Rose et al., who reported GPR (global 3%/1 mm pass criteria) failure at MFs of 4.9 MU/cGy while comparing dose distributions of SMC to film or TPS algorithm in SRT and SBRT plans. Rose et al. recommend limiting MFs to 3.0 MU/cGy for good agreement.^32^

Considering dosimetric differences between SMC and AAA off‐axis (Figures 6 and 7) we found them to be even more pronounced: GPR started failing 90% criteria at lower values of MFs compared to on‐axis, and the magnitude of the failure increased. For an LDT value of 10%, GPR failed at 6.0 MU/cGy, and for an LDT of 80%, GPR failed at 3.6 MU/cGy as compared to no failure with an LDT of 10% and failure at 6.8 MU/cGy with an LDT of 80% on‐axis. The average GPR between SMC and AAA off‐axis using an LDT of 80% was 67% compared to 86% on‐axis. Similar trends were observed in the comparison of SMC measurements to VMC++ calculations off‐axis, showing dramatically reduced agreement compared to on‐axis dose distributions.

Generally, PTV doses, measured by SMC in plans with highly modulated SRT fields were found to be lower than those calculated with Eclipse AAA and VIMC VMC++ (Figures 5 and 10). The lower reported dose by SMC is likely reflects the effect of 2.5 mm distance between SMC diodes for measurements in SRT fields, which consist of many small (smaller than 1 × 1 cm^2^) irregularly shaped sub‐fields.

Our results revealed a range of MFs where reasonable agreement was exhibited between the three dosimetric methods employed. Additionally, this study confirmed previous reports suggesting that SMC can be used for PSQA of stereotactic VMAT plans with MFs less than about 3.6 MU/cGy on and off‐axis, provided that appropriate LDT value is used. In such circumstances, SMC can be considered an efficient end‐to‐end PSQA tool.

Furthermore, our results highlighted the sensitivity of GPRs to selection of parameter values during analysis of SRT dose distributions. Notably, our analysis revealed that the 10% LDT may mask differences in high dose regions, leading to artificially high GPRs driven by diodes outside the PTV region. It is crucial to carefully consider the LDT, along with values of GPR, percent difference, and DTA criteria to ensure that errors are not overlooked during SRT VMAT quality assurance.

In light of these findings, we recommend using 80% LDT to closely encompass the PTV region for gamma analysis, as it proved to be considerably more sensitive for detecting meaningful dose discrepancies between SMC and other methods. This value is deemed appropriate as it is close to the region commonly used for dose prescription while avoiding the inclusion of steep dose gradient regions present in lower LDT values, such as 40%−60%. However, we acknowledge that under different circumstances, other LDT levels could be more appropriate, as long as essential regions of interest are included in the gamma analysis.

Our results emphasize the importance of carefully selecting LDT values tailored to the specific clinical context and regions of interest in gamma analysis.

## 5 CONCLUSIONS

Our study confirmed previous reports on increasing differences between calculated and measured dose with MF. The dosimetric differences were considerably higher for off‐axis fields in comparison to on‐axis fields. Interestingly, measured and calculated dose distributions started diverging, as reflected in the GPRs, once the MF exceeded a certain value. For on‐axis SRT VMAT fields, this value was approximately 4.6 MU/cGy, prompting us to recommend its clinical utilization as an upper limit. For off‐axis fields we recommend the value of about 3.6 MU/cGy as the upper threshold.

Our study also demonstrated LDT value of 80% to be more sensitive in gamma analysis for detecting dosimetric differences around the PTVs in SIMT SRT VMAT plans than commonly used 10% threshold value. Based on our findings, 80% LDT can be recommended where PTV coverage is the dominant clinical consideration. However, under different circumstances, other LDT levels could be appropriate as long as PTV is covered, and critical regions of interest are included in the gamma analysis. In these situations, it could be useful to adjust the LDT to focus on most clinically relevant regions of interest.

## AUTHOR CONTRIBUTIONS

Elena Timakova designed the research methodology, conducted data collection and analysis, and drafted the manuscript. Sergei Zavgorodni supervised the research, designed the research methodology, contributed to data interpretation, provided critical revisions, and approved the final version of the article. All authors read and approved the final manuscript.

## CONFLICT OF INTEREST STATEMENT

The authors declare no conflicts of interest.

## 1

Gamma pass rates as a function of modulation factor for 6 MV‐WFF energy beam, calculated with 20%−60% low dose threshold for VMAT plans with on‐axis and off‐axis PTVs.

On‐axis PTVs:

Off‐axis PTVs:
